# Sofosbuvir-based regimen is safe and effective for hepatitis C infected patients with stage 4–5 chronic kidney disease: a systematic review and meta-analysis

**DOI:** 10.1186/s12985-019-1140-x

**Published:** 2019-03-14

**Authors:** Mingshu Li, Jun Chen, Zhixiong Fang, Yi Li, Qian Lin

**Affiliations:** 1 Xiangya School of Public Health, Central South University, 110 Xiangya Road, Changsha, 410078 Hunan China; 2 Medical Affairs Department, Gilead Science, 179 Weifang Road, Shanghai, 200122 China; 3 Department of Liver Diseases, The Third People’s Hospital of Shenzhen, 29 Bulan Road, Shenzhen, 518114 Guangdong China; 4 Department of Infectious Disease, XiangTan City Central Hospital, 120 Heping Road, Xiangtan, 411100 Hunan China; 5 Department of Infectious Disease, The Second Xiangya Hospital of Central South University, 139 Renmin Road, Changsha, 410011 Hunan China

**Keywords:** HCV infection, Stage 4–5 chronic kidney disease, Sofosbuvir, Dose, Meta-analysis

## Abstract

**Background:**

Whether sofosbuvir is suitable for hepatitis C virus (HCV) infected patients with severe renal impairment is inconclusive. This systematic review aims to evaluate the safety and effectiveness of SOF-based regimen in the setting of stage 4 and 5 chronic kidney disease (CKD).

**Methods:**

We conducted a systematic literature search in PubMed, Web of Science, EMBASE and Google Scholar with searching strategy: (sofosbuvir OR Sovaldi OR Harvoni OR Epclusa OR Vosevi) AND (severe kidney impairment OR severe renal impairment OR end-stage renal disease OR dialysis OR renal failure OR ESRD OR renal insufficiency OR hepatorenal syndrome OR HRS). Sustained virological response (SVR12/24) rate and serious adverse event (SAE) rate with 95% confidence intervals were aggregated. Subgroup analysis was implemented to evaluate the impact of treatment strategy and patient characteristics.

**Results:**

Twenty-one studies met inclusion criteria, totaling 717 HCV infected patients with CKD stage 4 or 5 (58.4% on dialysis). Pooled SVR12/24 was 97.1% (95% CI 93.9–99.3%), and SAE rate was 4.8% (95% CI 2.1–10.3%). There was no significant difference at SVR12/24 (97.1% vs 96.2%, *p* = 0.72) or SAE rate (8.8% vs 2.9%, *p* = 0.13) between subgroups applying full or decreased dose of sofosbuvir. Cirrhotic and non-cirrhotic patients achieved comparable sustained virological response (RR 0.93, 95% CI 0.85–1.02). Four studies reported eGFR/serum creatinine pre- and post- treatment, with no significant modification.

**Conclusions:**

Our study suggests SOF-based regimen might be used safely and effectively in patients living with HCV infection/stage 4–5 CKD, with normal and reduced dose of sofosbuvir. Prospective and well-controlled trials are needed to confirm these findings.

**Trial registration:**

PROSPERO CRD42018107440.

**Electronic supplementary material:**

The online version of this article (10.1186/s12985-019-1140-x) contains supplementary material, which is available to authorized users.

## Background

Hepatitis C virus (HCV) infection and chronic kidney disease (CKD) are epidemically correlated and clinically challenging.

It’s estimated that 71 million people were chronically infected with HCV globally, and around 10% of them live with CKD [1, 2]. For patients with CKD, particularly patients receiving hemodialysis, the incidence of HCV is much higher than general population, ranging from 3 to 50% [3]. HCV infection significantly elevates renal disease progression, and clearing HCV has proved to reduce liver related mortality/complications as well as risk of HCV transmission, therefore, HCV cure is of great importance to the patients with dual burden [4–6].

Sofosbuvir, a nonstructural NS5B polymerase inhibitor, was approved in 2013 and has revolutionized HCV treatment, enhancing the cure bar to above 90% [7–9]. Sofosbuvir is mainly eliminated through renal pathway, and its use in patients with stage 4 and 5 CKD, defined according to KDIGO guidelines (GFR < 30 mL/min/1.73 m^2^), is not indicated in label [10, 11]. EASL Recommendations on Treatment of Hepatitis C 2018 suggested that sofosbuvir should be used with caution in patients with an eGFR< 30 ml/min/1. 73m^2^ or with end-stage renal disease, only if alternative treatment is not available [12]. AASLD Guidance: Recommendations for testing, managing and treating hepatitis C mentioned the safe and effective dose of sofosbuvir in persons with an eGFR< 30 ml/min have not been established. However, there is accumulating evidence on use of sofosbuvir-based regimen in those with an eGFR< 30 ml/min [13]. Therefore, we performed a systematic review and meta-analysis to evaluate the safety and effectiveness of SOF-containing therapy for this group of patients.

## Methods

### Literature search strategy

We followed PRISMA (Preferred reporting items for systematic review and meta-analyses) statement guidelines to conduct this study [14]. Systematic literature search in PubMed, EMBASE, Web of science, and Google Scholar was performed by two reviewers independently, without publishing date or language limitation. The searching strategy used was: (sofosbuvir OR Sovaldi OR Harvoni OR Epclusa OR Vosevi) AND (severe kidney impairment OR severe renal impairment OR end-stage renal disease OR dialysis OR renal failure OR ESRD OR renal insufficiency OR hepatorenal syndrome OR HRS). References listed in these literatures were also reviewed. Literature search was lastly updated on August 2018.

#### Inclusion criteria

Studies were included when following criteria was met:

1. Subject: HCV patients with stage 4 or 5 chronic kidney disease.
2. Intervention: SOF-based regimen.
3. Publication: articles, abstracts or letters.

#### Exclusion criteria

1. Patients with normal kidney function or early stage (1–3) CKD.
2. Number of enrolled patients or number of patients 12 weeks after treatment completion less than 10.
3. Insufficient data on SVR12/24, which is defined as undetectable HCV RNA 12 weeks (SVR12) or 24 weeks (SVR24) after treatment completion [12].
4. Insufficient HCV treatment combination information.
5. Case report.

### Data extraction

Two authors independently extracted data of study design, patients demographics and characteristics, treatment strategy, SVR12/24, renal function, SAE, discontinuation due to AE. Disagreement was resolved by consensus.

### Quality assessment

Newcastle-Ottawa scale (NOS) was applied to evaluate the quality and risk of bias of each study by two authors [15]. Studies were judged on three aspects, namely selection of study groups, the comparability of the groups and the exposure or outcome of participants. A score system was used for quality assessment in which a cumulative 7–9 score indicates high quality, 4–6 as fair quality.

### Data analysis

SVR12/24 rate, SAE rate were combined and assessed by fixed effect model/random effect model via R software. Heterogeneity among studies was evaluated by I^2^ index, with value > 50 implying substantial heterogeneity [16]. Fixed effect model was applied in the absence or minor heterogeneity, and random effect model was adopted for significant heterogeneity [17]. Comparison was made in subgroup analysis between studies that adopted full dose and decreased dose of sofosbuvir, RBV-containing and RBV-free regimen, studies that enrolled dialysis-dependent and dialysis-independent patients, as well as studies of different geographic origin. The relative risk (RR) with 95% CI was used to examine the impact of cirrhosis status to sustained virological response. We conducted sensitivity analysis to examine the robustness of primary findings. Publication bias was assessed by the Egger test for funnel plots asymmetry.

## Results

**Fig. 1:**
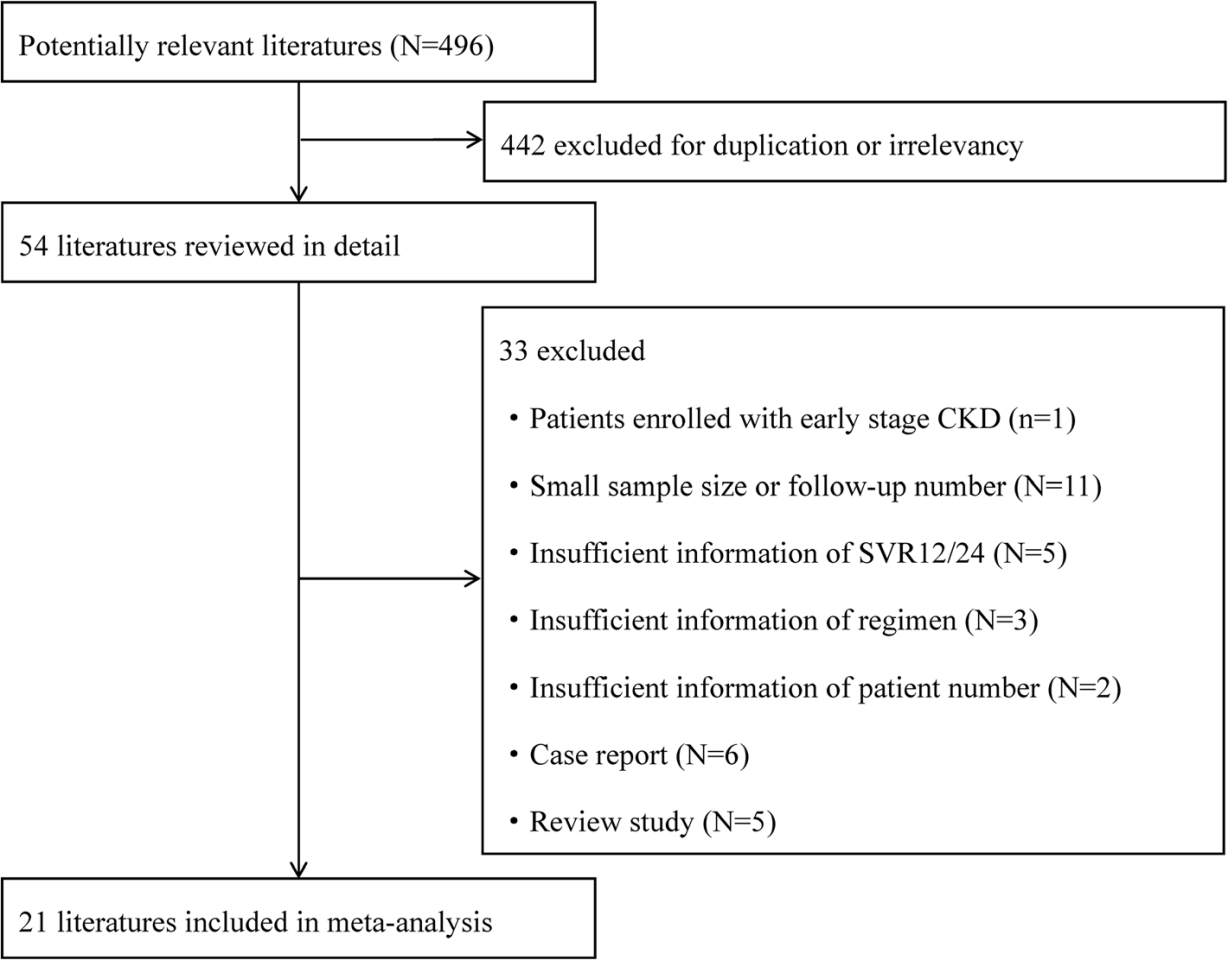
Flow diagram of literature search and selection

A total of 496 literatures were identified after preliminary search. Four hundred forty-two were excluded for duplication or irrelevancy. After further judgement by inclusion/exclusion criteria, final 21 studies were included for our review and meta-analysis, and IRB approval information were reported in 12 studies. Figure 1 shows the process of literature review and selection.

### Studies and patients’ characteristics

Twenty-one studies included manuscripts (*n* = 13), abstracts (*n* = 6) and letters (*n* = 2). All of them were prospective or retrospective cohort studies, with NOS score ranging 4–5 (two literatures scored 3). The absence of high quality is mainly due to absence of control arm. All of the studies met the inclusion criteria while one study enrolled patients with eGFR< 35 mL/min/1.73 m^2^ [18].

In total 717 patients were enrolled, including 419 (58.4%) hemodialysis or peritoneal dialysis recipients. Patients’ characteristics were shown in Table 1. Mean/median age ranged from 35 to 62 among studies. Eighteen studies provided genotype information, overall GT 1 was predominant genotype (67%), followed by GT 3 (20%) and GT2 (8%). Cirrhotic patient was eligible for most studies, and 4 studies included patients with decompensated cirrhosis. SOF-based regimen included: SOF + SMV ± RBV, SOF + PR, SOF + RBV, SOF + DCV ± RBV and SOF/LDV ± RBV, with varied administration of sofosbuvir: 400 mg daily (QD), 200 mg QD, 400 mg/48 h, 400 mg three times a week (TIW). Dose of sofosbuvir during treatment was rarely adjusted, except 2 patients increased from 200 mg QD to 400 mg QD after 4–6 weeks, and 3 reduced dosing due to sepsis, digestive discomfort or headache.

**Table 1:** Characteristics of studies and patients

Studies	Geographical origin	No. of patients	No. of dialysis recipients	History of Cirrhosis (%)	mean/median baseline RNA	Genotype	SOF-based regimen	Dose of SOF	SVR12/24 (PP)	NOS score
Aggarwal (2017) [19]	USA	14	14	20% (F3,F4)	8,375,588.6 IU/ML	GT1–60%, GT2–6.7%, GT3–20%, GT4–13.3%	SOF + SMV, SOF + RBV, SOF/LDV ± RBV, SOF + PR, SOF + DCV 12–24 W	200 mg QD	92.8% 13/14	4
Akhil (2018) [26]	India	22	22	NA	2,642,495 IU/ML	GT1–63.63%, GT3–27.27%, GT4–9%	SOF + RBV 12 W	400 mg QD	80% 16/20	4
Beinhardt (2016) [27]	Austria	10	10	40% (30% decompensation)	6.1 ± 0.8 log IU/ML	GT1a-20%, GT1b-40%, GT3a-20%, GT4–20%	SOF + PR, SOF + SMV, SOF + DCV, SOF + RBV 12–24 W	400 mg QD	90% 9/10	4
Bera (2017) [20]	India	25	25	20%	6.4 ± 0.57 log IU/ML	GT3–72%, GT1–24%, GT4–4%	SOF + DCV 12–24 W	400 mg/48 h	100% 16/16	4
Bhamidimarri (2015) [31]	USA	15	12	60%	9.7 × 10^6^ IU/ML	GT1a-67%, GT1b-33%	SOF + SMV 12–24 W	200 mg QD or 400 mg/48 h	87% 13/15	4
Butt (2108) [45]	USA	137	NA	NA	NA	NA	SOF/LDV ± RBV 12–16 W	400 mg QD	95% 103/108	3
Choudhary (2017) [21]	India	16	16	12.50%	7 (5–8) log IU/ML	GT1–69%, GT3–25%, GT-6%	SOF + PR, SOF + DCV ± RBV12 W	400 mg/48 h	80% 8/10	4
Desnoyer (2016) [32]	France	12	12	83%	6.59 (6.13–6.86) log IU/ML	GT1–92% GT2–8%	SOF + SMV, SOF + DCV, SOF/LDV, SOF + RBV 12–24 W	400 mg QD or 400 mg TIW	83% 10/12	5
Dumortier (2017) [18]	France	50	35	54%	2,603,063 IU/ML	GT1–56%, GT2–12%, GT3–10%, GT4–18%, GT5–4%	SOF + RBV, SOF + PR, SOF + DCV ± RBV, SOF + SMV ± RBV 12–24 W	400 mg QD or 400 mg/48 h or 400 mg TIW	91% 43/47	5
Taneja (2018) [22]	India	65	54	32.3%(9% decompensation)	1.65 × 10^6^ (1.2 × 10^3^–1.73 × 10^8^) IU/ML	GT1–65%; GT2–1%, GT3–34%	SOF + DCV 12- 24w	200 mg QD	100% 65/65	5
Goel (2018) [23]	India	41	31	12%	5.9 (4.12–9.9) log IU/ML	GT3–54%, GT1–42%, GT4–5%	SOF + DCV 12–24 W	200 mg QD	100% 36/36	4
Yingli (2017) [24]	China	33	33	NA	1.7–7.8 log IU/ML	GT1b-21%, GT2a-73%, GT2a + 1b-6%	SOF + DCV	200 mg QD	100% 33/33	4
Lawitz (2017) [33]	USA and New Zealand	18	0	11%	NA	GT1a-78%, GT1b-22%	SOF/LDV 12 W	400 mg QD	100% 18/18	4
Manoj (2018) [28]	India	64	11	NA	NA	NA	SOF + RBV, SOF/LDV, SOF + DCV 12–24 W	400 mg QD	100% 64/64	5
Mehta (2018) [46]	India	38	38	NA	5.75 (5.05–6.36) log IU/ML	GT1a-42%, GT1b-58%	SOF + DCV, SOF/LDV 12 W	400 mg QD or 400 mg/48 h	86.8% 33/38	5
Nazario (2017) [29]	USA	41	38	49%	NA	GT1a-66%; GT2–2%, GT3–2%	SOF + SMV, SOF/LDV, SOF + DCV 12–24 W	400 mg QD	100% 41/41	3
Saab (2017) [30]	USA	12	12	NA	30,499,500 ± 29,655,754 IU/ML	GT1a-42%, GT1b-25%, GT2–17%, GT1–17%	SOF + RBV, SOF/LDV ± RBV	400 mg QD	70% 7/10	4
Saxena (2015) [47]	USA	18	5	75%	NA	NA	SOF + PR, SOF + RBV, SOF + SMV ± RBV	400 mg QD	85% 11/13	5
Singh (2017) [34]	USA	36	30	27.8% (16.7% decompensation)	9.9 × 10^5^ IU/ML	G1–72%, G3–22%, G4–5%	SOF/LDV, SOF + DCV 12 W–24 W	400 mg QD	97.2% 35/36	4
Surendra (2018) [25]	India	21	21	0	NA (63% > 800,000 IU/ML)	GT1a-63%, GT1b-37%	SOF/LDV 12 W	400 mg/48 h	100% 19/19	5
Cox-North (2017) [35]	USA	29	NA	44%(14% decompensation)	NA	GT1–72%, GT2–7%, GT3–17% GT6–4%	SOF/LDV ± RBV, SOF + DCV ± RBV, 8-24 W	400 mg QD	100% 28/28	4

*SMV* simeprevir, *PR* Peg-interferon/ribavirin, *DCV* daclatasvir, *LDV* ledipasvir

### Sustained virological response

**Fig. 2:**
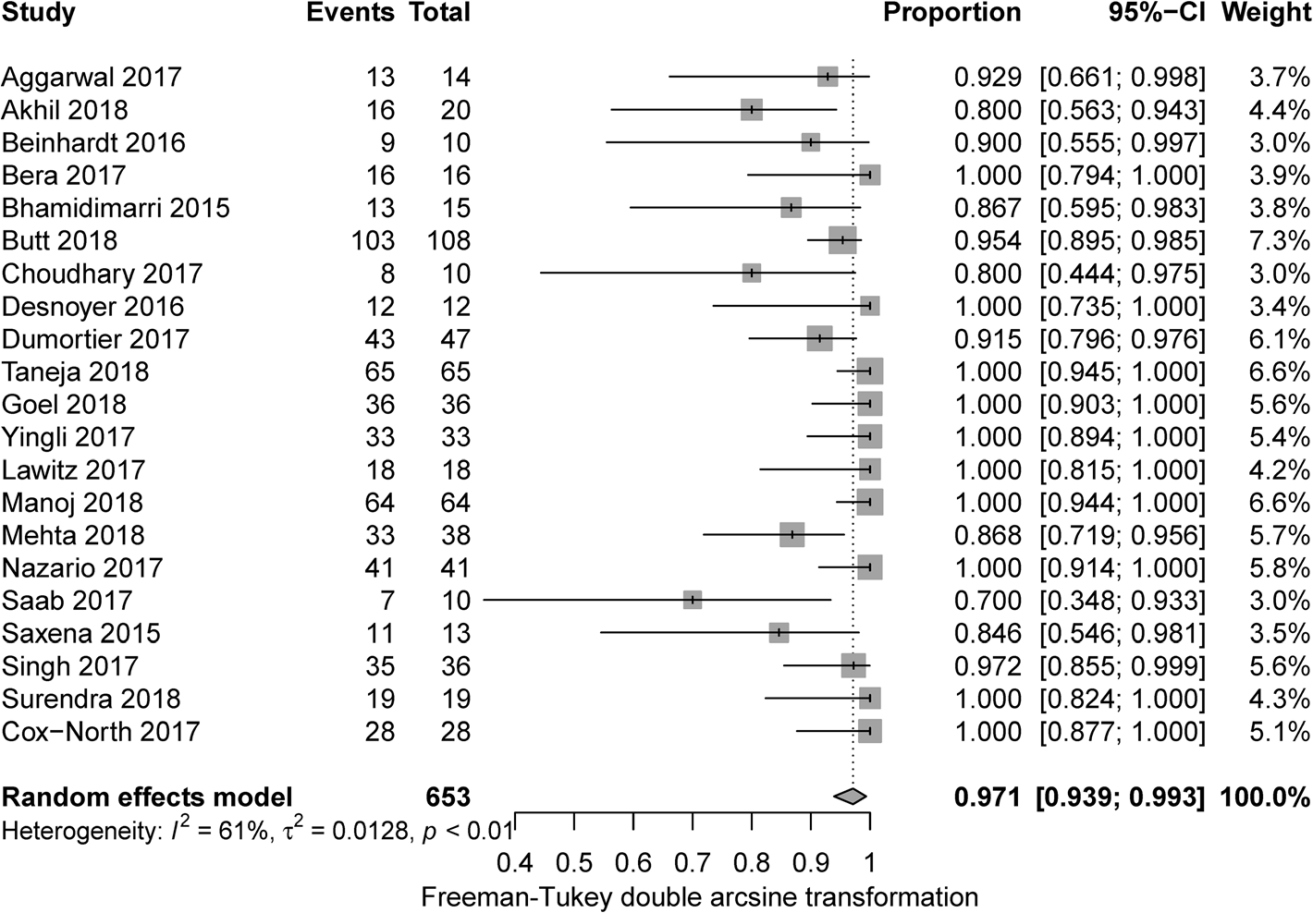
Forest plots showing the results of pooled SVR12/24

Per protocol (PP) analysis set was applied for sustained virological response analysis. The pooled SVR12/24 rate was 97.1% with random effect model (95% CI 93.9–99.3%, I^2^ = 61%) (Fig. 2). By aggregating dialysis-dependent patients (*n* = 306) and the others who were not on dialysis (*n* = 88) based on data available, we found significant difference of SVR12/24 between these two groups (95.1% vs 100%, *p* = 0.019). (Additional file 1: Figure S1).

**Fig. 3:**
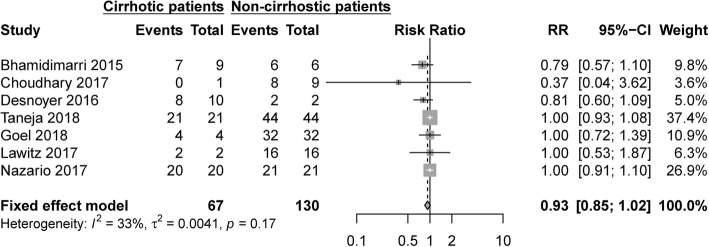
Forest plots showing the results of meta-analysis comparing SVR12/24 in patients with cirrhosis versus patients without cirrhosis

Studies with full sofosbuvir dose (400 mg QD) or decreased dose were compared for subgroup analysis. The difference between full dose (97.1, 95% CI: 92.1–99.9%) and decreased dose (96.2, 95%CI: 88.3–100%) was not significant (*p* = 0.72) (Additional file 2: Figure S2). Studies that applied single therapy of decreased-dose of sofosbuvir (i.e. 200 mg QD or 400 mg/48 h or 400 mg TIW) were further selected and compared, concluding that 200 mg QD and 400 mg/48 h demonstrated similar treatment effect on total population (100% vs 97.7%, *p* = 0.30) (Additional file 3: Figure S3) [19–25]. For patients on dialysis, SVR12/24 was also comparable among varied doses of sofosbuvir (*p* = 0.25) (Additional file 4: Figure S4) [19–22, 24–30]. Ten studies adopted RBV-free regimen (SOF/LDV, SOF + DCV, SOF + SMV) with pooled SVR12/24 99.1% (95% CI: 96.2–100%), higher than that of studies using RBV-containing therapy (94.0%,95 CI: 87.5–98.6%) (*p* = 0.035). Seven studies provided sufficient data for comparison between patients with or without cirrhosis [21–23, 29, 31–33]. Non-cirrhotic patients trended to have higher sustained virological response, while there was no significant difference between these two subgroups (RR 0.93, 95% CI 0.85–1.02, I^2^ = 33%) (Fig. 3).

Most of the studies in this analysis originated from Asia and Norther America, and the pooled SVR12/24 was comparable among different regions (*p* = 0.15) (Additional file 5: Figure S5).

### Serious adverse event

**Fig. 4:**
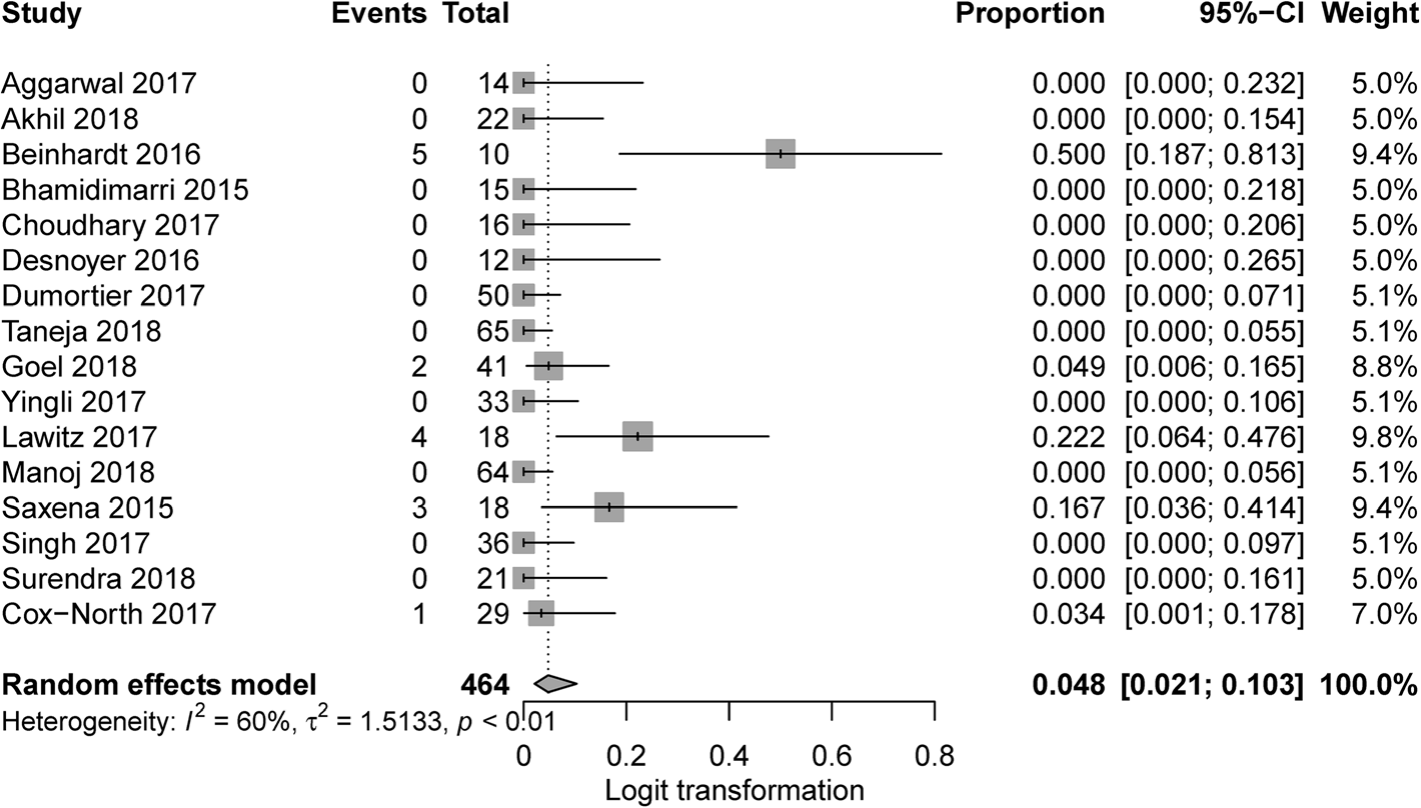
Forest plots showing the results of pooled SAE rate

Information of SAE rate was provided in 16 studies, of which the pooled incidence was 4.8% (95% CI 2.1–10.3%, I^2^ = 60.0%) (Fig. 4). SAE occurred in 5 studies, and 4 provided case description (Table 2). Subgroup analysis was conducted for studies with full dose and decreased-dose sofosbuvir, resulting no significant difference (8.8% vs 2.9%, *P* = 0.13).

**Table 2:** Serious adverse events

Study	No. of patients	No. of patients/events with SAE	Description of SAE (comments from investigator)
Beinhardt (2016) [27]	10	5 (5/5 on dialysis)	1 pt.: recurring peritonitis
			1 pt.: renal anemia
			1 pt.: graft failure after orthotopic liver transplantation (LOT)
			1 pt.: cirrhosis due to HCV recurrence after OLT
			1 PT: pneumonia
Goel (2018) [23]	41	2 (0/2 on dialysis)	1 pt.: acute mild pancreatitis after renal transplantation
			1 pt.: worsening of ascites
Lawitz (2017) [33]	18	4 (0/4 on dialysis)	1 pt.: acute kidney injury and noncardiac chest pain
			1 pt.: dehydration and hypotension
			1 pt.: acute renal failure
			1 pt.: hypotension and synocope (no SAEs were considered related to study drug)
Saxena (2015) [47]	18	3	NA
Cox-North (2017) [35]	29	1 (1/1 on dialysis)	1 pt.: cardiac event (unable to draw any conclusions about the safety of SOF regimens in those with underlying cardiac disease)

### Change in renal function

Four studies reported the dynamic of kidney function [18, 22, 34, 35], with detailed data before and after treatment (Table 3). Generally, eGFR was stable during treatment, and 2 cases reported discontinuation from hemodialysis due to eGFR improvement [35].

**Table 3:** Kidney function before and after treatment

Study	No. of patients	Mean/Median eGFR (mL/min/1. 73m^2^)/ Serum Creatinine (mg/dL) at baseline	Mean/Median eGFR (mL/min/1. 73m^2^)/ Serum Creatinine (mg/dL) after treatment	*P*-value/Comments from investigator
Dumortier (2017) [18]	15 non-HD patients	eGFR 29.0 (20–34)	eGFR 27.0 (17–38)	In non-HD patients, median eGFR was not significantly modified during treatment.
Taneja (2018) [22]	11 pre-dialysis patients	eGFR 24.84 ± 3.96	eGFR 24.39 ± 3.63	0.82
		Creatinine2.52 ± 0.35	Creatinine 2.56 ± 0.36	0.81
Singh(2017) [34]	36	eGFR 12.02 ± 6.89	eGFR 12.33 ± 6.10	0.77
Cox-North (2017) [35]	NA patients not receiving dialysis	eGFR 22.2 creatinine 3.1	eGFR 20 creatinine 3.3	1.0

### Sensitivity analysis

SVR12/24 of 13 manuscripts (PP analysis set) was aggregated, with pooled result of 97.0% (95% CI: 92.4–99.7%, I^2^ = 66%). 90.3% SVR12/24 (95% CI: 83.4–95.6%, I^2^ = 84%) was resulted in intention to treat (ITT) analysis set for 21 studies. Not reaching SVR12 time point and lost follow-up were two major reasons for relative lower result in ITT analysis set.

### Publication bias

*P* value of Egger test for funnel plots asymmetry was not significant for ITT analysis set (0.537), and bordered significance level for PP analysis set (0.0498).

## Discussion

Patients living with HCV infection and end-stage renal disease (ESRD) are special population for HCV treatment. Although current guidelines recommend the first-line therapies as elbasvir/grazoprevir, glecaprevir/pibrentasvir, paritaprevir/ ritonavir/ombitasvir/dasabuvir (PrOD) [12, 13], unmet medical needs still exist at some cases (e.g. comorbidity of advanced liver disease, non-GT1 genotype) and when other therapies are not available. In these circumstances, sofosbuvir might be applied after weighing risk and benefit. In vivo, sofosbuvir undergoes intra-hepatic metabolism to form the pharmacologically active uridine analog triphosphate (GS-461203), which eventually results in ultimate metabolite GS-331007 via dephosphorylation [36]. Sofosbuvir and GS331007 are mainly eliminated through kidney. Compared with subjects of normal renal function, area under the curve (AUC_0-inf_) of sofosbuvir and GS-331007 is 171 and 451% higher for patients with eGFR< 30 ml/min (not receiving hemodialysis) [11]. Desnoyer examined plasma concentrations of sofosbuvir (full dose) and GS331007 on hemodialysis patients and concluded they did not accumulate throughout the treatment course and between hemodialysis sessions [32]. Whether sofosbuvir and its metabolite accumulate in ESRD patients who are not on dialysis needs to be answered by further study.

Our meta-analysis included 21 studies, with a total of 717 patients. Pooled SVR12/24 was satisfying (97.1%), similar or higher than that of non-SOF-based therapies [37–39]. Patients who were on dialysis also achieved a SVR12/24 as high as 95.1%. Although it was lower than that of patients without dialysis, we assume it might not necessarily be the case given limited number of dialysis-free patients in our sub-analysis. Further well-designed RCT are needed to conclude whether effect of sofosbuvir is influenced by dialysis. Aggregated SAE rate was 4.8%, slightly higher than that in HCV infected patients with normal renal function [8, 9], which is reasonable since the patients involved in our meta-analysis had quite a few safety risk factors: old age, severe renal dysfunction, liver/renal transplant recipient, and advanced liver fibrosis. In another meta-analysis, SAE rate was 12.1% for direct-acting antivirals-based antiviral therapies in HCV/Stage 4–5 CKD patients [40]. Common SAEs included renal failure, cirrhosis complications, indicating special attention is needed on renal and liver function during treatment (Table 2).

Treatment strategy of SOF-containing therapy has been under broad discussion. Many physicians explored unconventional dose of sofosbuvir for safety concern, although there is no established pharmacokinetics profile for administration at 200 mg QD, 400 mg/48 h or 400 mg three times a week. Our subgroup analysis suggests that regimen with full and decreased dose of sofosbuvir might be both considerable at acceptable SAE rate (8.8% vs 2.9%) and high SVR12/24 (97.1% vs 96.2%). We assume that sofosbuvir could be alternatively applied at a lower dose as half as normal or at a frequency extended to once every 2 days without compromising its efficacy significantly. While administering three times a week might not be optimal, given that median terminal half-lives of sofosbuvir and its metabolite GS331007 were 0.4 and 27 h (healthy subjects), and possible low SVR12/24 (60% reported in very small sample size) [32]. Ribavirin should be used with caution, considering higher risk of anemia for RBV-containing regimen than RBV-free treatment. Anemia was the most frequently reported AE (6 to 43.7%) in studies of our review, and RBV was included in almost all of these studies (8/9). Manoj reported that 65.4% (17/26) patients in SOF + RBV group developed anemia, and 30% had to discontinue ribavirin [28]. What is worth mentioning is that we found RBV-containing regimen reached lower SVR12/24. One of the reasons is likely to be tolerability issue of ribavirin, another reason might be potent combination drug with sofosbuvir in most RBV-free regimen, 9 studies applied SOF/LDV or SOF + DCV, and 7 achieved 100% SVR12/24.

Kidney function deterioration is a concern for sofosbuvir usage. On one hand, there are few case reports proposed the correlation of nephrotoxicity and SOF-based therapy [41, 42], on the other hand, several large retrospective cohorts conclude SOF-based regimen does not introduce higher acute kidney injury for HCV patients compared to SOF-free treatment [43, 44]. Studies that reported renal function in our review had generally stable eGFR and serum creatinine during treatment. Two SAE cases of AKI and acute renal failure were considered not related to study drug.

There are some limitations to our study. First, we could not perform subgroup analysis according to HCV genotype, CKD stage for lack of enough information. Second, geographical origin of studies enrolled is mainly USA and India, and that limits the representativeness of our analysis. Third, all of the studies were observational studies without control group, and most studies were of medium quality. Heterogeneity was substantial which might attribute to varied sample size and treatment therapy. Furthermore, some newly approved SOF-based therapy (e.g. SOF/VEL) was not included in this analysis for lack of evidence and analysis time frame. The factors above compromise the quality of this review. Prospective and well-controlled studies are expected in near future to provide more robust evidence.

## Conclusions

This systematic review and meta-analysis evaluated SOF-based therapy for HCV infected patients with comorbidity of stage 4–5 CKD. Data from this study suggests satisfying sustained virological response and tolerance. For treatment strategy, both full and decreased dose of sofosbuvir could be appropriate. Caution is still needed at clinical practice.

## Additional files

Additional file 1: **Figure S1.** Forest plots showing the results of subgroup analysis result of SVR12/24 in dialysis-dependent patients and patients not receiving dialysis. (PDF 6 kb)

Additional file 2: **Figure S2.** Forest plots showing the results of subgroup analysis result of SVR12/24 in studies applied full dose and decreased dose of sofosbuvir. (PDF 6 kb)

Additional file 3: **Figure S3.** Forest plots showing the results of subgroup analysis result of SVR12/24 in studies applied 200 mg QD sofosbuvir or 400 mg/48 h sofosbuvir. (PDF 5 kb)

Additional file 4: **Figure S4.** Forest plots showing the results of subgroup analysis result of SVR12/24 in dialysis-dependent patients applying different doses of sofosbuvir. (PDF 6 kb)

Additional file 5: **Figure S5.** Forest plots showing the results of subgroup analysis result of SVR12/24 in patients originated from Asia, North America and Europe. (PDF 7 kb)

## Abbreviations

AKI: Acute kidney injury
CKD: Chronic kidney disease
DCV: Daclatasvir
ESRD: End-stage renal disease
GFR: Glomerular filtration rate
HCV: Hepatitis C virus
HRS: Hepatorenal syndrome
ITT: Intention to treat
KDIGO: Kidney Disease Improving Global Outcomes
LDV: Ledipasvir
MELD: Model for end-stage liver disease
NOS: Newcastle-Ottawa scale
PP: Per protocol
PR: Peg-interferon/ribavirin
PRISMA: Preferred reporting items for systematic reviews and meta-analyses
PrOD: Paritaprevir/ ritonavir/ombitasvir/dasabuvir
RBV: Ribavirin
SAE: Serious adverse event
SMV: Simeprevir
SOF: Sofosbuvir
SVR: Sustained virological response
TIW: Three times a week
