# Supplementary figures and images for: Sofosbuvir-based regimen is safe and effective for hepatitis C infected patients with stage 4–5 chronic kidney disease: a systematic review and meta-analysis
Source: Virol J. 2019 Mar 14;16:34. doi: 10.1186/s12985-019-1140-x (PMC6419462; doi:10.1186/s12985-019-1140-x)

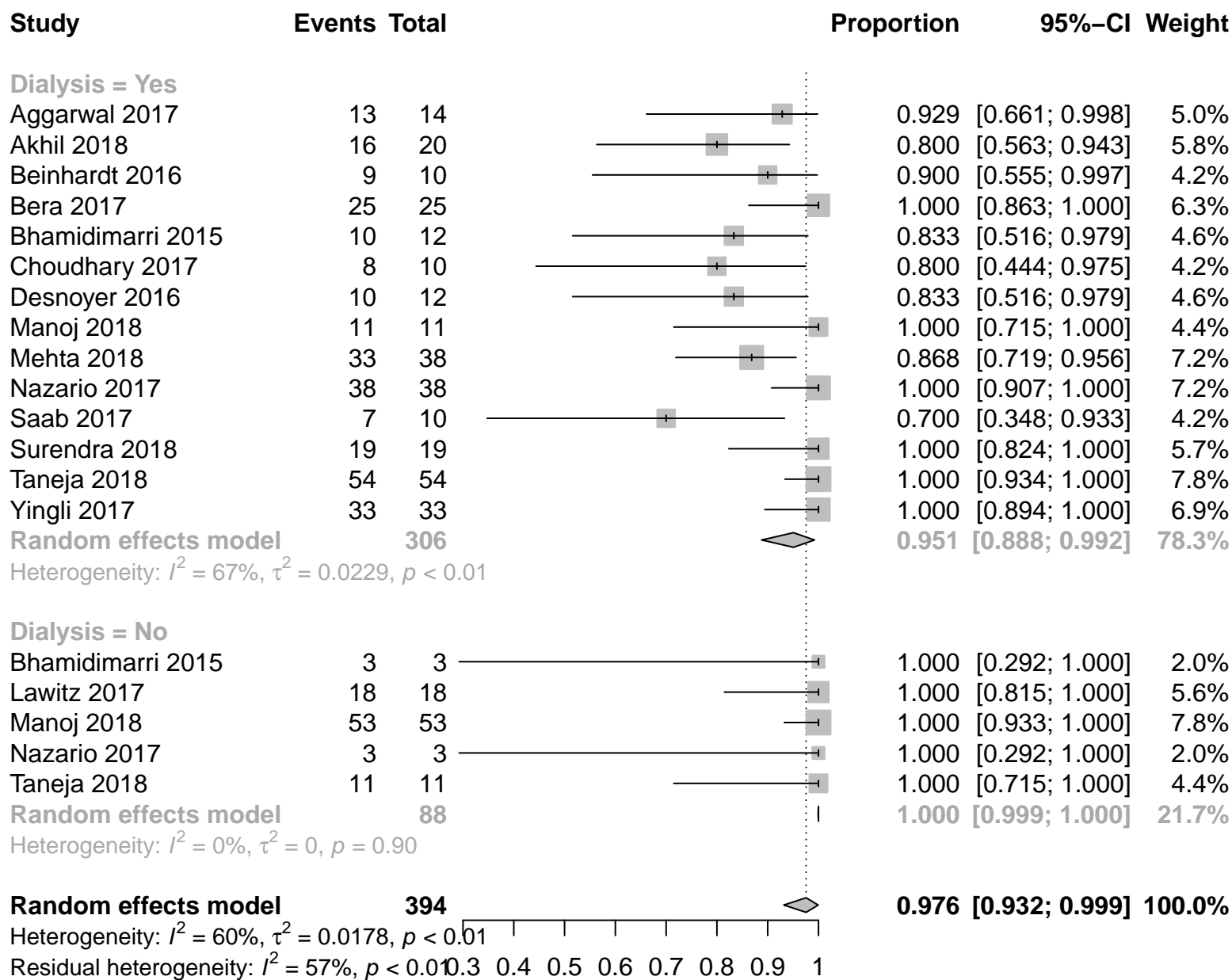

Supplement: Supplementary file 1 — Figure S1. Forest plots showing the results of subgroup analysis result of SVR12/24 in dialysis-dependent patients and patients not receiving dialysis. (PDF 6 kb) [file 12985_2019_1140_MOESM1_ESM.pdf]

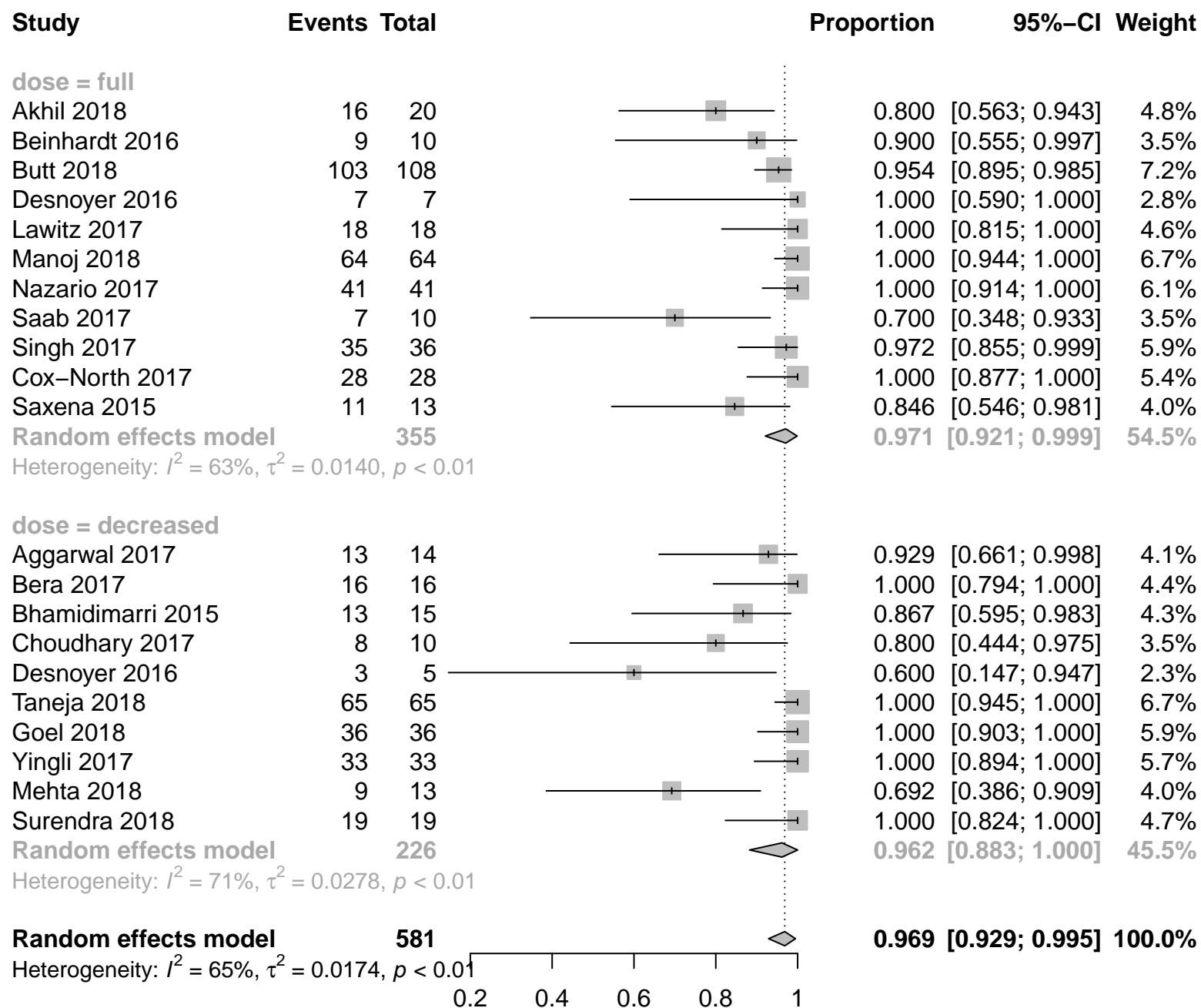

Supplement: Supplementary file 2 — Figure S2. Forest plots showing the results of subgroup analysis result of SVR12/24 in studies applied full dose and decreased dose of sofosbuvir. (PDF 6 kb) [file 12985_2019_1140_MOESM2_ESM.pdf]

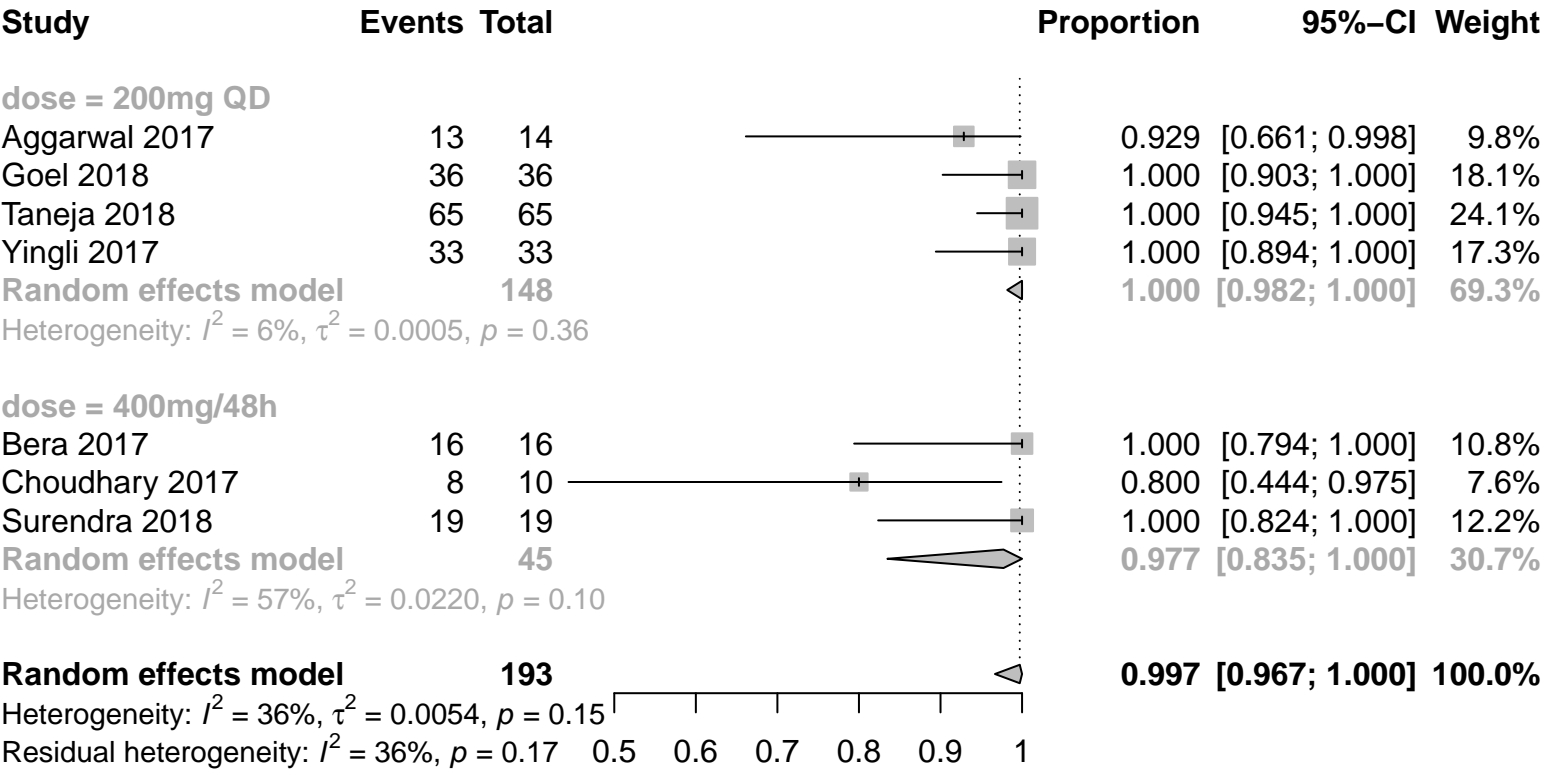

Supplement: Supplementary file 3 — Figure S3. Forest plots showing the results of subgroup analysis result of SVR12/24 in studies applied 200 mg QD sofosbuvir or 400 mg/48 h sofosbuvir. (PDF 5 kb) [file 12985_2019_1140_MOESM3_ESM.pdf]

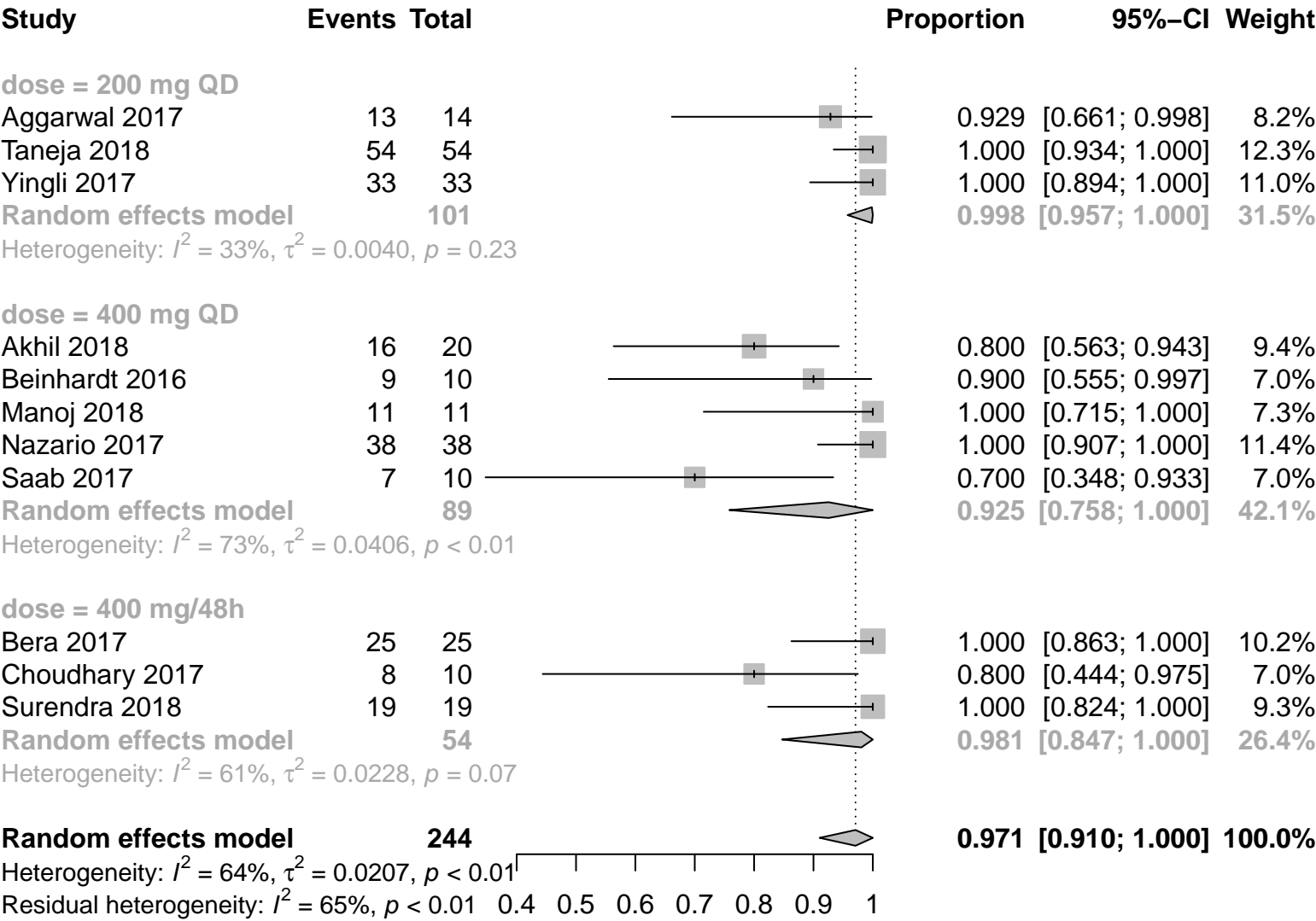

Supplement: Supplementary file 4 — Figure S4. Forest plots showing the results of subgroup analysis result of SVR12/24 in dialysis-dependent patients applying different doses of sofosbuvir. (PDF 6 kb) [file 12985_2019_1140_MOESM4_ESM.pdf]

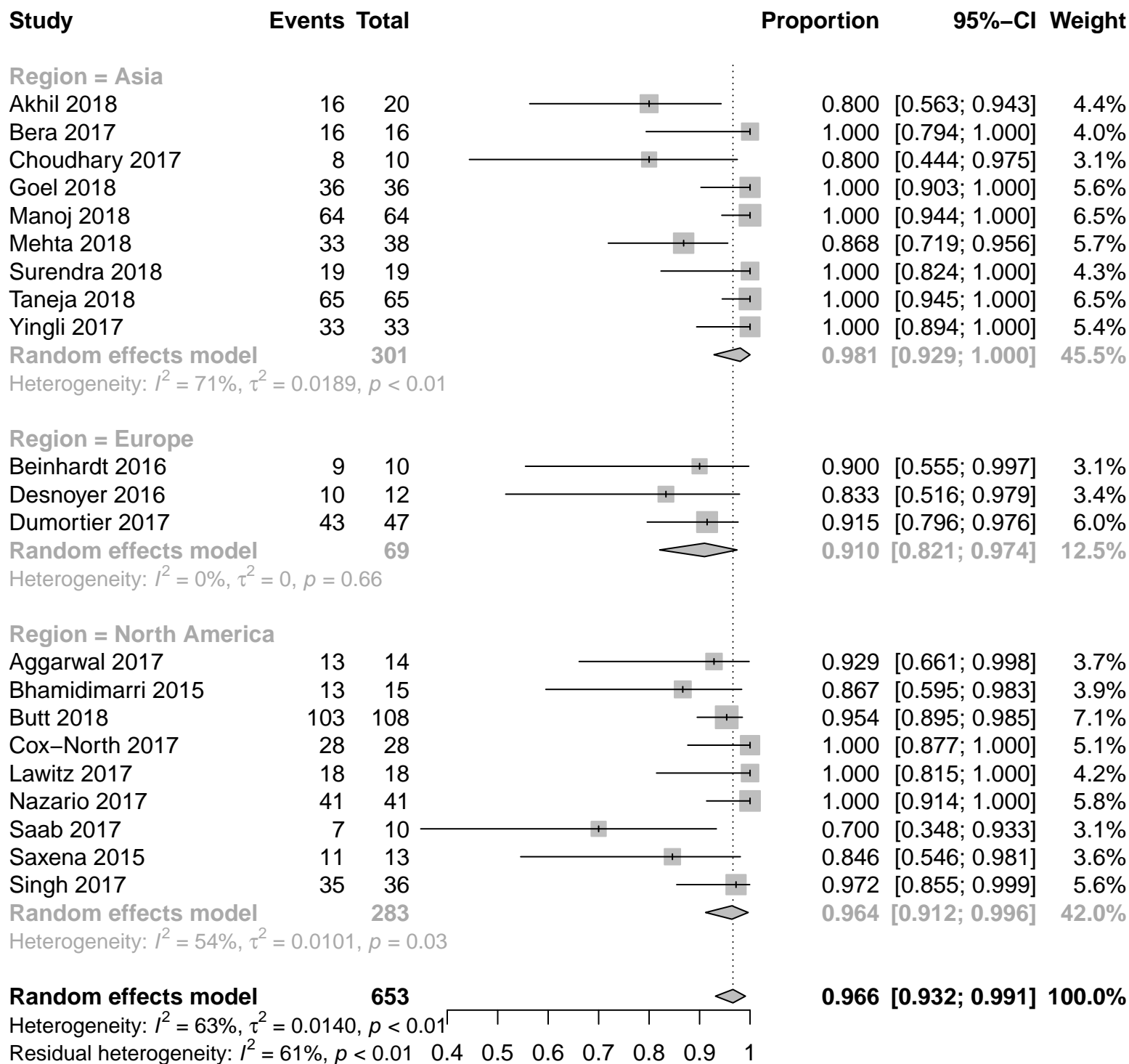

Supplement: Supplementary file 5 — Figure S5. Forest plots showing the results of subgroup analysis result of SVR12/24 in patients originated from Asia, North America and Europe. (PDF 7 kb) [file 12985_2019_1140_MOESM5_ESM.pdf]
